# Expanding the Antiviral Spectrum of Scorpion-Derived Peptides Against Toscana Virus and Schmallenberg Virus

**DOI:** 10.3390/pathogens14070713

**Published:** 2025-07-19

**Authors:** Rosa Giugliano, Carla Zannella, Roberta Della Marca, Annalisa Chianese, Laura Di Clemente, Alessandra Monti, Nunzianna Doti, Federica Cacioppo, Valentina Iovane, Serena Montagnaro, Simona De Grazia, Massimiliano Galdiero, Anna De Filippis

**Affiliations:** 1 Department of Experimental Medicine, University of Campania Luigi Vanvitelli, 80138 Naples, Italy; rosa.giugliano@unicampania.it (R.G.); carla.zannella@unicampania.it (C.Z.); roberta.dellamarca@unicampania.it (R.D.M.); annalisa.chianese@unicampania.it (A.C.); laura.diclemente@unicampania.it (L.D.C.); massimiliano.galdiero@unicampania.it (M.G.); 2 Department of Veterinary Medicine and Animal Production, University of Naples Federico II, 80137 Naples, Italy; serena.montagnaro@unina.it; 3 Institute of Biostructures and Bioimaging (IBB), National Research Council (CNR), 80131 Naples, Italy; alessandra.monti@ibb.cnr.it (A.M.); nunzianna.doti@cnr.it (N.D.); 4 Department of Health Promotion, Mother and Child Care, Internal Medicine and Medical Specialties “G. D’Alessandro”, University of Palermo, 90127 Palermo, Italy; federica.cacioppo@unipa.it (F.C.); simona.degrazia@unipa.it (S.D.G.); 5 Department of Agricultural Sciences, University of Naples Federico II, 80055 Portici, Italy; valentina.iovane@unina.it; 6 Complex Operative Unit of Virology and Microbiology, University Hospital of Campania “Luigi Vanvitelli”, 80138 Naples, Italy

**Keywords:** pantinins, scorpion-derived peptides, Bunyavirales, Toscana virus, Schmallenberg virus, emerging viral infections, antivirals, animal viruses

## Abstract

Toscana virus (TOSV) and Schmallenberg virus (SBV) are arthropod-borne viruses from the *Bunyaviricetes* class, posing significant human and animal health threats. TOSV, endemic to the Mediterranean region, is a notable human pathogen detected in various animals, suggesting potential zoonotic reservoirs. SBV emerged in Europe in 2011, affecting ruminants and causing reproductive issues, with substantial economic implications. The rapid spread of both viruses underscores the need for novel antiviral strategies. Host defense peptides (HDPs), particularly those derived from scorpion venom, are gaining attention for their antiviral potential. This study investigated pantinin-1 and pantinin-2 for their inhibitory activity against TOSV and SBV by plaque reduction assay, tissue culture infective dose (TCID_50_) determination, and the analysis of *M* gene expression via qPCR. Both peptides exhibited potent virucidal activity, with IC_50_ values of approximately 10 µM, depending on the specific in vitro cell model used. Additionally, the selectivity index (SI) values were favorable across all virus/cell line combinations, with particularly optimal results observed for pantinin-2. In human U87-MG neuronal cells, both peptides effectively blocked TOSV infection, a critical finding given the virus’s association with neurological conditions like encephalitis. The strong efficacy of these peptides against these viruses underscores the broader applicability of venom-derived peptides as promising antiviral agents, particularly in the context of emerging viral pathogens and increasing resistance to conventional therapeutics. Further studies are needed to optimize their antiviral potency and to assess their safety in vivo using animal models.

## 1. Introduction

The *Bunyaviricetes* class is a large group of arthropod-borne viruses responsible for several diseases affecting both humans and animals. Within the order Bunyavirales, several families are present, comprising more than 600 species, some of which have raised particular concern due to various outbreaks in recent years. Considerable diseases caused by Bunyavirales are Crimean-Congo hemorrhagic fever [1,2,3], Rift Valley fever [4,5], Oropouche fever [6,7,8,9], Sandfly fever [10,11,12,13,14], and Hantavirus hemorrhagic fever with renal syndrome [15,16]. These diseases are characterized by symptoms such as fever, hemorrhagic fever, meningitis, encephalitis, and renal failure. Transmission occurs through the bites of mosquitoes, flies, and ticks, or contact with rodents, to vertebrate reservoir hosts. These types of viruses significantly threaten the global public and veterinary health. The growing incidence of arboviral diseases is accelerated by climate change, increased travel and trade, and changes in land use and animal husbandry practices, all of which expand the habitat of vectors and facilitate virus transmission.

Among arboviruses, Schmallenberg virus (SBV) is an orthobunyavirus, first identified in Germany in 2011 and quickly spreading across Europe, causing significant impacts on livestock and posing a serious issue for veterinary public health [17]. The virus induces a broad spectrum of clinical manifestations: in adult animals, symptoms are typically mild, including fever, diarrhea, and reduced milk production; in offspring, however, SBV can cause severe outcomes, such as premature birth, stillbirth, or the birth of severely malformed animals, often leading to death [18]. Overall, the seroprevalence of SBS among domestic ruminants is estimated at 49%, with the highest rates observed in cattle (59%), followed by sheep (37%) and goats (18%) [19,20]. The impact of SBV on the livestock industry is considerable. A study conducted in France estimated the economic impact of SBV at the farm level, with financial losses ranging from 0.6% to 63% of the gross margin, depending on the selected scenario and the farming system evaluated. This situation has sparked growing interest in identifying effective therapeutic interventions and preventive measures across various European nations. Similarly, another virus of significant importance is Toscana virus (TOSV), a member of the *Phlebovirus* genus, which is endemic in the Mediterranean basin, parts of Europe, and the Middle East. It has been estimated that the number of TOSV in Italy during 2022–2023 was 2.6 times higher than in 2016–2021 [21]. TOSV is primarily transmitted to humans and other vertebrates by *Phlebotomus* spp. sandflies. In humans, TOSV infection is often asymptomatic or accompanied by a mild, febrile illness. However, due to its marked neurotropism, the virus can also lead to severe neurological manifestations, including meningitis and encephalitis. While humans are considered accidental hosts, a wide range of vertebrates, including livestock species, dogs, cats, and bats, have been implicated as potential reservoirs or amplification hosts [12,22,23,24].

Therefore, a One Health approach is essential for understanding the ecology and transmission dynamics of arboviruses and developing novel therapeutic strategies to restrict their impact on human and animal health [25]. To date, there are no vaccines or effective antivirals against Bunyavirales infections. One of the key limitations in controlling these infections lies in the three-segmented RNA genome of Bunyavirales, which facilitates genetic reassortment and recombination events, potentially leading to the emergence of novel viral strains with altered virulence, host range, or transmissibility [26,27,28,29].

A promising strategy in the ongoing search for effective antiviral therapeutics involves the study of host defense peptides (HDPs), which are components of the innate immune system of many organisms [30]. Among these, peptides derived from the venom of scorpions have garnered increasing attention [31,32,33]. Several peptides derived from scorpion venom have demonstrated strong antiviral activity against other arboviruses, such as Zika virus (ZIKV) and Dengue virus type 2 (DENV-2) [34,35,36]. At present, no specific drugs are available against SBV or TOSV. Galidesivir (BCX4430), an adenosine nucleoside analogue, has exhibited broad-spectrum activity against various arboviruses, including yellow fever virus, ZIKV, and Rift Valley fever virus (RVFV), and it is currently under clinical evaluation [37]. In this study, to identify potential therapeutic agents against SBV and TOSV, we investigate the antiviral properties of two scorpion-derived HDPs, pantinin-1 and pantinin-2. Both peptides demonstrate significant virucidal activity, with half-maximal inhibitory concentration (IC_50_) values in the micromolar (μM) range. Future investigations should aim to elucidate their mechanisms of action, evaluate their in vivo efficacy, and assess their suitability for clinical translation as antiviral therapeutics.

## 2. Materials and Methods

### 2.1. Reagents

Chemicals were from commercial sources and used without further purification unless otherwise stated. Reagents and solvents, including acetonitrile (CH_3_CN), dimethylformamide (DMF), dichloromethane (DCM), tetrahydrofuran (THF), and methanol (CH_3_OH), were purchased as sym-collidine, N,N-diisopropylethylamine (DIPEA), piperidine, acetic anhydride (Ac2O), tri-isopropylsilane (TIS) and trifluoroacetic acid (TFA) from Merck (Milan, Italy). Protected amino acids, the resin, and coupling agents including HATU (2-(1H-7-Azabenzotriazol-1-yl)-1,1,3,3-tetramethyl-uronium-hexafluorphosphate), OxymePure^®^ (ethyl 2-cyano-2-(hydroxyimino)acetate), sym-collidine, and N-N′ diisopropylcarbodiimide (DIC) used for peptide synthesis were from IRIS Biotech GmbH (Marktrewitz, Germany).

### 2.2. Peptide Synthesis and Characterization

Pantinin-1 (single-letter sequence: GILGKLWEGFKSIV-NH_2_) and pantinin-2 (IFGAIWKGISSLL-NH_2_) were synthesized, as C-terminal amidated variants, by solid-phase synthesis methodology, using oxime/DIC and HATU/sym-collidine as coupling agents and an automated SYRO I synthesizer (Biotage, Uppsala, Sweden), according to optimized protocols developed in our laboratory [38]. A Fmoc-Rink AM amide resin (loading 0.56 mmol/g) and standard Fmoc amino acids were used in all syntheses. Peptides were cleaved from the resins by treatment with a mixture of trifluoroacetic acid (TFA)/tri-isopropylsilane (TIS)/water (95:2.5:2.5, *v*/*v*/*v*) for 3 h at room temperature. Crude peptides were precipitated in cold diethyl ether, dissolved in a H_2_O/CH_3_CN mixture (75:25, *v*/*v*), and lyophilized. After lyophilization, peptides were purified by reversed-phase HPLC (RP-HPLC) on a WATERS 2545 system with a UV/Vis detector (Waters, Milan, Italy) using a Jupiter 5 µm C18 (300 Å 150 × 21.2 mm, Ea) semipreparative column (Phenomenex, Bologna, Italy), applying a CH_3_CN linear gradient (5–80% in 10 min) containing 0.1% TFA at a flow rate of 15 mL/min and monitoring the absorbance at 214 nm. The collected fractions were lyophilized. Peptide purity and identity were confirmed by liquid chromatography–mass spectrometry (LC-MS) analysis, using an Agilent 1260 Infinity II LC/MSD system (Milan, Italy) with a Waters xBridge C18 column (5 µm, 2.1 × 50 mm), applying a linear CH3CN gradient (10–80% in 10 min) containing 0.005% TFA, with a flow rate of 0.2 mL/min. All peptides achieved purity levels above 95% with a yield of 80% (Figures S1 and S2).

### 2.3. Cell and Virus Growth

Baby hamster kidney cells (BHK-21; ATCC CCL-10), U-87 MG (ATCC HTB-14), canine fibrosarcoma cells (A-72 cells; ATCC CRL-1542), and Crandell feline kidney cells (CRFK; ATCC CCL-94) were used. All cell lines were maintained in Dulbecco’s Modified Eagle Medium (DMEM) with 4.5 g/L glucose (cod. D5796, Microtech, Naples, Italy), supplemented with a 100 IU/mL penicillin/streptomycin solution (cod. A001A, Himedia, Naples, Italy) and 10% fetal bovine serum (FBS; cod. RM10432, Microtech, Naples, Italy). All cell lines were maintained below passage 10 and routinely tested for the absence of mycoplasma contamination. The two viruses, TOSV (ISS Phl.3) and SBV, were propagated in the BHK-21 cell monolayer. TOSV was acquired from the European Virus Archive Global (EVAg, Marseille, France), while SBV was kindly provided by Professor Giuseppe Iovane (University Federico II, Naples, Italy). All experiments involving infectious viruses were conducted under Biosafety Level 2 (BSL-2) conditions following institutional biosafety regulations. The use of animal-derived cell lines complied with national and European guidelines for the ethical use of biological materials.

### 2.4. Cytotoxicity

BHK-21, U-87 MG, A-72, and CRFK cells were seeded in 96-well plates at an initial density of 2 × 10^4^ cells per well and incubated for 24 h at 37 °C. Then, serial dilutions of peptides ranging from 3 to 200 µM were added to the cell monolayer and incubated for 24 h at 37 °C. Cytotoxicity was evaluated by adding 5 μL of methylthiazolyldiphenyl-tetrazolium bromide (MTT; cod. M2003, Sigma-Aldrich, St. Louis, MO, USA) to each well. After 4 h of incubation, the plate was emptied, and formazan crystals were solubilized with dimethyl sulfoxide (DMSO; 100%). Cell viability was calculated by measuring absorbance at 570 nm using a microplate reader [39]. The negative control (CTRL−, toxicity control) consisted of cells treated with DMSO, while the positive control (CTRL+, viable cells) included untreated cells.

### 2.5. Antiviral Activity

The antiviral activity of peptides against TOSV and SBV was assessed using two different methods: (1) the plaque assay, which is based on lysis plaque counts following the death of infected cells, and (2) tissue culture infective dose (TCID_50_), a widely used method based on the cytopathic effect (CPE), utilized for viruses that do not form plaques. The antiviral activity was assessed using various cell lines, including BHK-21, U-87 MG, A72, and CRFK.

#### 2.5.1. Plaque Assay

BHK-21 and U-87 MG cells (1.4 × 10^5^ cells/well) were seeded in 24-well plates and incubated at 37 °C for 24 h. The confluent cell monolayer was infected with TOSV and treated with serial dilutions of peptides by varying the timing of their addition. As previously reported [40,41], four different experiments were conducted: (i) Co-treatment: peptides and virus at a multiplicity of infection (MOI) of 0.01 were inoculated on the cell monolayer in a 1:1 ratio simultaneously for 1 h. (ii) Virus pre-treatment: virus at 0.1 MOI and peptides were pre-incubated for 1 h at 37 °C. Then, the mixture (peptide and virus) was diluted in FBS-free DMEM and dispensed onto the cells for 1 h, so that the peptides were used at non-active concentrations, while the virus was applied at the MOI of 0.01. (iii) Cell pre-treatment: the cell monolayer was pre-treated for 1 h with peptides and then infected with the virus at 0.01 MOI for 1 h. (iv) Post-treatment: cells were infected at 0.01 MOI and, after 1 h, the infected monolayer was treated with peptides. In all the cases, after the time of virus adsorption (1 h), cells were washed with citrate buffer and covered with DMEM supplemented with 1 mL of 5% carboxymethylcellulose (CMC; Sigma-Aldrich, Darmstadt, Germany) to restrict viral spread from the initial site of infection. After 24 h of incubation, cells were washed twice with PBS 1X, then fixed with 4% formaldehyde and stained with 0.5% crystal violet (CV) to visualize the plaque zones. The positive control (CTRL+) and negative control (CTRL−) were represented by oreochromicin-1 (25 µM) and untreated infected cells, respectively. Finally, the plaques were counted by using the ImageJ tool version 1.54p (https://imagej.net, accessed on 20 March 2025), and the percentage (%) of viral infection was calculated by comparing the number of plaques in infected cells treated with peptides to those in untreated infected cells.

#### 2.5.2. Tissue Culture Infective Dose Assay

To evaluate the action of peptides against SBV, the TCID_50_ method was performed. In brief, cells (2 × 10^4^ cells/well) were seeded in a 96-well plate and incubated for 24 h. The following day, the same experimental schemes mentioned above were conducted (co-treatment, virus pre-treatment, cell pre-treatment, and post-treatment). In detail, 10-fold serial dilutions of the viral suspension (from 10^−1^ to 10^−8^) were prepared in complete medium and added to the cells in quadruplicate wells for each dilution. After viral adsorption, fresh medium was added to each plate well and incubated for 48 h at 37 °C. The cells were fixed with 4% formaldehyde and stained with 0.5% CV to observe CPE under a light microscope, and the Reed and Munch method was used to calculate the result of TCID_50_/mL, based on the dilution at which 50% of the wells exhibited CPE [42].

### 2.6. Real-Time Quantitative PCR (qPCR)

BHK-21 cells (1.4 × 10^5^ cells/well) were seeded in 24-well plates and incubated at 37 °C for 24 h. The infection was performed following the virus pre-treatment scheme, as mentioned above. After the viral adsorption, cells were covered with fresh medium and incubated at 37 °C for 48 h. Then, total RNA was collected using TRIzol reagent (Thermo Fisher Scientific, Waltham, MA, USA) and reverse-transcribed into cDNA using the 5× All-In-One RT MasterMix Kit (Applied Biological Materials, Richmond, BC, Canada). The expression of the viral segment M of SBV was detected by quantitative real-time PCR (qRT-PCR). Cycle threshold (Ct) values of the target gene in infected, treated cells were compared to those in infected, untreated controls and normalized to the housekeeping gene glyceraldehyde 3-phosphate dehydrogenase (GAPDH). The primer sequences used for the GAPDH gene were forward (5′-CCTTTCATTGAGCTCCAT-3′) and reverse (5′-CGTACATGGGAGCGTC-3′), and for the *M* gene they were forward (5′-TCAATTCAGCAAGTAACATACAATGG-3′) and reverse (5′-CGTGGTCTGTCTTGGTTGATG-3′). Finally, the relative quantification was calculated with the 2^−ΔΔCt^ method [43].

### 2.7. Calculation of Selectivity Index

The evaluation of the selectivity index (SI) is essential for assessing the safety range between the dose required to achieve the antiviral effect and the dose that produces cytotoxicity. The SI was calculated considering the data related to efficacy and cytotoxicity (CC_50_/IC_50_). Higher SI values indicate greater antiviral efficacy.

### 2.8. Statistics

All tests were performed in triplicate and expressed as mean ± standard deviation (SD). Graphs and statistical analysis were generated using GraphPad Prism version 9.5.1 for macOS (GraphPad Software, San Diego, CA, USA, www.graphpad.com, accessed on 5 February 2025). The 50% cytotoxic concentration (CC_50_) and the 50% inhibitory concentration (IC_50_) were calculated by nonlinear regression analysis based on dose–response curves generated from at least three independent experiments.

## 3. Results

### 3.1. Pantinins’ Cytotoxicity on Different Cellular Models

**Figure 1:**
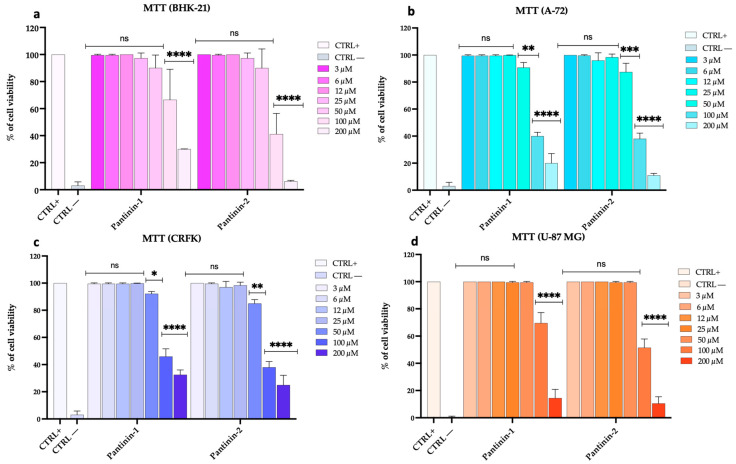
Assessment of cell viability using the MTT test following 24 h of peptide treatment. (**a**) BHK-21 cell line, (**b**) A-72 cell line, (**c**) CRFK cell line, and (**d**) U-87 MG cell line. The positive control (CTRL+) consisted of untreated cells, whereas the negative control (CTRL−) was represented by DMSO (100%). Two-way ANOVA with Dunnett’s multiple comparisons test was performed. Statistical analysis is related to CTRL+. **** *p* < 0.0001, *** *p* < 0.0002, ** *p* = 0.0021, * *p* = 0.0332, and ns = not significant.

We conducted a comprehensive analysis to assess the potential cytotoxic effects of pantinin-1 and pantinin-2 on various animal and human cell lines, including BHK-21, A-72, CRFK, and U-87 MG cells, via the MTT assay (Figure 1).

Our data demonstrated that both peptides exhibited marked cytotoxicity when applied at the highest concentration of 200 μM, indicating a potential threshold for cellular tolerance. As the concentration was reduced to 100 μM, the toxicity decreased considerably. Notably, at concentrations of 50 μM and below, neither pantinin-1 nor pantinin-2 induced any detectable cytotoxicity in the tested cell lines. Similar results were observed after 48 h of treatment in BHK-21 cells (Figure S3).

### 3.2. Inhibitory Effects of Pantinins on SBV Replication

**Figure 2:**
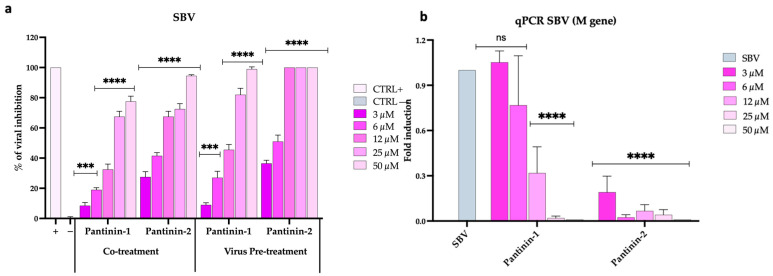
Antiviral activity of pantinins during SBV infection in BHK-21 cells. (**a**) Evaluation of antiviral activity using the TCID_50_ method in co-treatment and virus pre-treatment assays. Oreoch-1 peptide was used as a positive control (CTRL+), and infected cells were used as a negative control (CTRL−). (**b**) Quantitative real-time PCR investigation of *M* gene expression following virus pre-treatment with pantinin-1 and pantinin-2. Two-way ANOVA with Dunnett’s multiple comparisons test was performed. **** *p* < 0.0001, *** *p* < 0.0002, and ns = not significant.

We evaluated the antiviral potential of pantinin-1 and pantinin-2 in reducing SBV infection using two different assays: the reduction of CPE measured by TCID_50_, and quantitative analysis through qPCR (Figure 2).

In the initial assay, both peptides demonstrated the highest activity under two conditions: (1) when co-administered with SBV onto a BHK-21 cell monolayer (Figure 2a, co-treatment), and (2) when used to pre-treat the virus before cell inoculation (Figure 2a, virus pre-treatment). Specifically, pantinin-2 exhibited greater antiviral efficacy than pantinin-1 under both treatment conditions, with IC_50_ values of 7.16 μM versus 18.4 μM in the co-treatment assay, and 4.8 μM versus 12.8 μM in the virus pre-treatment assay. No antiviral effect was observed for either peptide in the other two treatment conditions described in the Section 2, namely, cell pre-treatment (when the cell monolayer was pre-treated with peptides and then infected) and post-treatment (when the infected cell monolayer was treated with peptides), suggesting that the peptides did not act on the cell surface or within the infected cell, respectively (Figure S4a,b). Overall, our findings indicate that pantinin-1, and particularly pantinin-2, are endowed with antiviral potency against SBV infection, likely through interference with viral particles during the early phase of infection.

We then evaluated the two pantinins’ ability to modulate SBV infection by quantifying the expression levels of the viral *M* gene using qPCR (Figure 2b). The gene can code for the glycoprotein C (Gc) and glycoprotein N (Gn), exposed on the surface of the SBV, besides the non-structural protein NSm. Accordingly, the absence of viral gene expression at the highest concentrations of pantinin-1 and pantinin-2 indicates a complete inhibition of transcription. As the peptide concentrations decreased, a dose-dependent increase in viral gene expression was observed.

### 3.3. Inhibitory Effects of Pantinins on TOSV Replication

**Figure 3:**
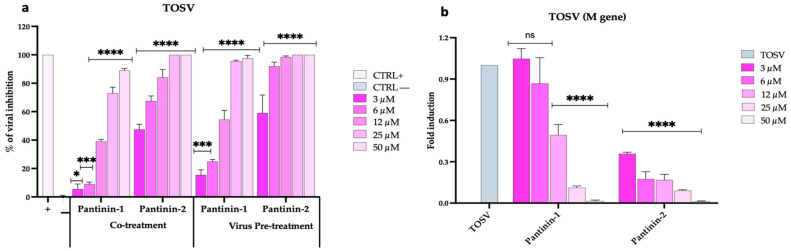
Antiviral activity of pantinins during TOSV infection in BHK-21 cells. (**a**) Evaluation of antiviral activity by the plaque reduction assay in co-treatment and virus pre-treatment assays. Oreoch-1 peptide was used as a positive control (CTRL+), and infected cells were used as a negative control (CTRL−). (**b**) Quantitative real-time PCR investigation of *M* gene expression following virus pre-treatment with pantinin-1 and pantinin-2. Two-way ANOVA with Dunnett’s multiple comparisons test was performed. **** *p* < 0.0001, *** *p* < 0.0002, * *p* = 0.0332, and ns = not significant.

We then extended the evaluation of the antiviral activity of pantinins against TOSV using a broad panel of both animal and human cell lines. First, we assessed the effect of pantinins in the BHK-21 cell monolayers (Figure 3), an established in vitro model commonly used to culture TOSV [44,45], in co-treatment and virus pre-treatment conditions, and by qPCR.

As shown in Figure 3a, pantinins exhibited their strongest antiviral activity in the co-treatment and virus pre-treatment assays, consistent with the results previously observed against SBV (Figure 2a). Similarly, the inhibitory effect was more pronounced for pantinin-2 compared to pantinin-1 under both experimental conditions, showing an IC_50_ of 3 μM versus 16.3 μM in the co-treatment assay, and 2.5 µM versus 11 μM in the virus pre-treatment assay (Figure 3a). We then confirmed these data using molecular analysis via qPCR. A virus pre-treatment assay was performed, RNA was extracted and reverse-transcribed, and the expression of the viral gene, TOSV M, was detected (Figure 3b). At the highest concentration of both pantinins (50 μM), viral gene expression was completely suppressed. A dose-dependent inhibition of *M* gene transcription was observed with decreasing concentrations of both peptides, although transcription levels remained markedly low across all concentrations of pantinin-2.

### 3.4. Inhibitory Effects of Pantinins Against SBV and TOSV Infection in Animal Cell Models

**Figure 4:**
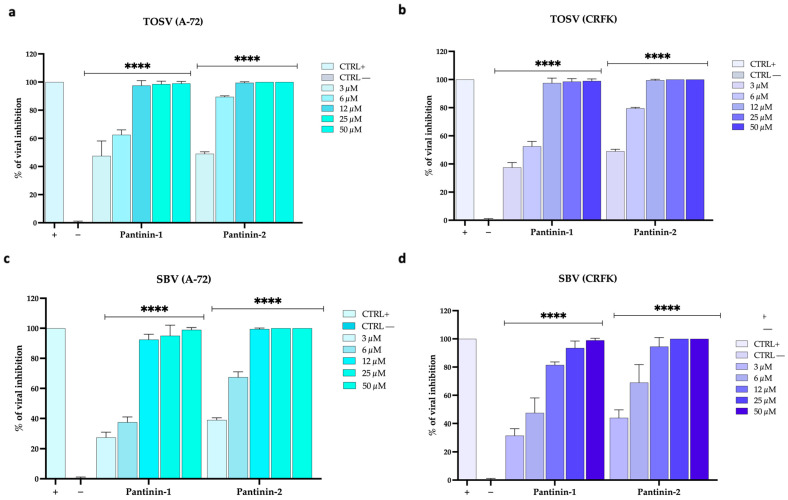
Antiviral activity of pantinins in animal cells by TCID_50_. (**a**,**b**) Antiviral activity against TOSV in virus pre-treatment in A-72 and CRFK cells. (**c**,**d**) Antiviral activity of pantinins against SBV in virus pre-treatment in A-72 and CRFK cells. Oreoch-1 peptide was used as a positive control (CTRL+), and infected cells were used as a negative control (CTRL−). Two-way ANOVA with Dunnett’s multiple comparisons test was performed, **** *p* < 0.0001.

Another important observation was that pantinins were also able to prevent SBV and TOSV infection in animal cell models, such as A-72 (canine cells) and CRFK (feline cells; Figure 4).

First, we evaluated the ability of both viruses to replicate in these cellular systems and observed rapid replication kinetics in both animal cell models. Second, a strong antiviral effect was noted when TOSV (Figure 4a,b) or SBV (Figure 4c,d) were pre-treated with pantinins and the mixture was subsequently titrated on A-72 cells and CRFK cells (virus pre-treatment assay). As previously described, pantinins—particularly pantinin-2—are endowed with antiviral potency. Specifically, pantinin-2 exhibited IC_50_ values of 3 μM against TOSV infection in A-72 and CRFK cells. In contrast, pantinin-1 displayed slightly lower anti-TOSV efficacy, with IC_50_ values of 3.8 μM in A-72 cells and 5 μM in CRFK cells.

### 3.5. Pantinins Inhibit TOSV Infection in U-87 MG Neuronal Cells

**Figure 5:**
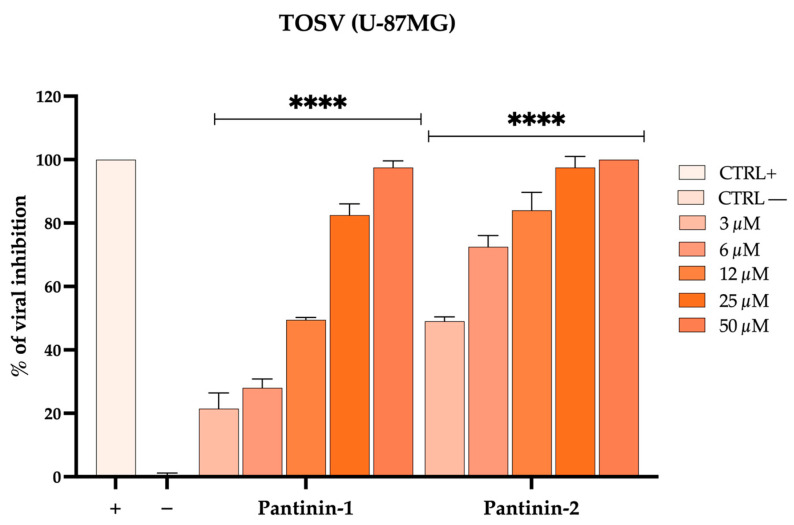
Antiviral activity of pantinins against TOSV in neuronal cells by the plaque assay. Antiviral activity of pantinins against TOSV in virus pre-treatment on U-87MG cells. Oreoch-1 peptide was used as a positive control (CTRL+), and infected cells were used as a negative control (CTRL−). Two-way ANOVA with Dunnett’s multiple comparisons test was performed, **** *p* < 0.0001.

We aimed to investigate the ability of pantinins to reduce TOSV infection in U-87 MG cells. Neuronal cells were exposed to viral particles pre-treated with pantinin-1 or pantinin-2 at concentrations ranging from 3 to 50 μM (virus pre-treatment assay). After 24 h post-infection (hpi), viral plaques were counted, and the % of infection inhibition was calculated (Figure 5).

Our results demonstrate that pantinins are also capable of reducing viral infection in neuronal cells, employed here as an in vitro model to mimic the virus’s neurotropic properties. For clarity, all CC_50_, IC_50_, and SI values of pantinin-1 and pantinin-2 against SBV and TOSV in the various cell types used in this study are summarized in Table 1.

## 4. Discussion

The present study expands the antiviral efficacy of scorpion-derived AMPs, pantinin-1 and pantinin-2, against two clinically relevant viruses: SBV and TOSV. These viruses share some common characteristics, including transmission through vectors and their ability to infect a range of vertebrate hosts [17,18,23,46]. Regarding TOSV infection, numerous reports indicate that the virus can be transmitted to humans, potentially leading to severe neurological diseases, due to its neurotropic nature [47,48,49,50]. In contrast, there is no evidence of SBV transmission from ruminants to humans. However, concerns about the potential zoonotic spillover of SBV have been raised due to the segmented nature of its RNA genome, which may facilitate genetic reassortment with other viruses, as already observed in several bunyaviruses [51,52,53,54]. In addition, different studies found anti-TOSV antibodies in many dogs and cats, making them sentinel indicators of virus circulation due to high seropositivity [23,55,56,57,58].

Unfortunately, our arsenal against these infections remains limited, as no specific antiviral drugs or licensed vaccines are currently available for either virus. This highlights the urgent need to develop novel therapeutic and preventive strategies.

Our previous studies have demonstrated that pantinins are capable of inhibiting in vitro infections caused by animal herpesviruses, suggesting for the first time their potential as antiviral agents. We also demonstrated their antibacterial effect against several bacterial strains, including both Gram-positive and Gram-negative species, and analyzed their serum stability [59]. In the present work, we aimed to investigate whether their spectrum of activity could also be extended to riboviruses belonging to the *Bunyaviricetes* class. Our findings demonstrated that both pantinins possessed potent antiviral activity with an IC_50_ of approximately 10 μM against SBV and TOSV, and in different cell models, particularly when administered in co-treatment or virus pre-treatment conditions (Figure 2a, Figure 3a, Figure 4 and Figure 5), indicating a direct interaction with viral particles as the primary mode of action. Notably, neither peptide showed antiviral activity in post-infection or cell pre-treatment assays (Figure S4), indicating their effects were virucidal rather than inhibiting cellular entry mechanisms or intracellular replication pathways. Pantinin-2 showed a higher antiviral effect, as evidenced by the lower IC_50_ values compared to those of pantinin-1 (Table 1).

This difference in efficacy may reflect variations in amino acid sequence or peptide structure, which could influence their binding affinity to the viral membrane. For instance, pantinin-2 exhibited greater hydrophobicity and a more pronounced amphipathic character, forming a more compact helix that inserts more effectively into membranes. In our recent study, we demonstrated that, despite their comparable primary sequences, pantinins show distinct conformational properties. Specifically, the C-terminal region of pantinin-2 (from Ile9 to Leu12) showed a higher propensity for the transient helical secondary structure than pantinin-1 when peptides were in aqueous solution. On the other hand, in membrane-mimetic environments, both peptides adopted well-defined α-helix structures [59]. Similarly, Xia et al. reported that the scorpion venom peptide BmKn2 and its derivative BmKn2-T5, both of which share a typical amphiphilic structure with α-helix, showed significant inhibitory activity at 10 µg/mL against enterovirus 71 (EV71) and that BmKn2-T5 showed a lower cell cytotoxicity than BmKn2 [60]. Recently, the Botcl peptide showed antiviral activity against Newcastle disease virus (NDV) with an IC_50_ of 0.69 μM and CC_50_ > 55 µM [61].

Our qPCR data (Figure 2b and Figure 3b) validated these observations, demonstrating that treatment with pantinins effectively suppressed viral gene expression. For SBV (Figure 3b), the downregulation of the *M* gene, which encodes key surface glycoproteins, further supports the hypothesis that pantinins block the viral infection, possibly by disrupting the integrity of the viral envelope. Similar inhibitory patterns were observed with TOSV (Figure 2b), where pantinins also inhibited the expression of the M viral gene, reinforcing the conclusion of a strong antiviral effect.

Importantly, the pantinins exhibited minimal cytotoxicity at high concentrations, indicating their potential as safe antiviral agents, as shown by the SI value, which was very favorable for each virus/cell line and was especially higher in pantinin-2 (Table 1).

The ability of these peptides to inhibit TOSV and SBV in both human neuronal and various animal-derived cell lines also highlights their broad host range application, which is a valuable result considering the zoonotic nature of many viruses belonging to the *Bunyaviricetes* class. The observed activity against neurotropic TOSV in U87-MG cells is particularly promising, as it suggests a potential role in managing virus-associated neurological disorders, such as encephalitis. Our findings support the knowledge that pantinin-1 and pantinin-2 possess strong antiviral activity also against Bunyavirales infections, highlighting their broader potential as scaffolds for the design of novel antiviral agents, especially in the context of emerging viral threats.

However, this study has certain limitations. The results are based solely on in vitro experiments, and further in vivo investigations are necessary to assess the peptides’ efficacy, stability, and safety in animal models. Moreover, the precise mechanisms underlying their antiviral action remain not fully understood. Although the data suggest envelope-targeting activity, no direct mechanistic assays were performed. Future studies, including transmission electron microscopy (TEM), surface plasmon resonance (SPR), or liposome leakage assays, should focus on elucidating the molecular interactions between pantinins and viral components, which will be crucial for the rational design and optimization of peptide-based antiviral strategies.

## Figures and Tables

**Table 1:** CC_50_, IC_50_, and SI values of pantinin-1 and pantinin-2 against SBV and TOSV in different cell lines.

	Pantinin-1 (µM)					Pantinin-2 (µM)				
		SBV		TOSV			SBV		TOSV	
	CC_50_	IC_50_	SI	IC_50_	SI	CC_50_	IC_50_	SI	IC_50_	SI
BHK-21	136.6	12.8	10.7	11	12.4	100.8	4.8	21	2.5	40.3
A-72	93.6	6	15.6	3.8	24.6	90.7	4	22.7	3	30.2
CRFK	93.5	5.8	16.1	5	18.7	82.2	3.7	22.2	3	27.4
U-87	114.3	-	-	12	9.5	82	-	-	3	27.3

## Data Availability

The data presented in this study are available upon request from the corresponding author. Authors can confirm that all relevant data are included in the article.

## Supplementary Materials

The following supporting information can be downloaded at: https://www.mdpi.com/article/10.3390/pathogens14070713/s1, Figure S1: Pantinin-1 characterization; Figure S2: Pantinin-2 characterization; Figure S3: Evaluation of cell viability using the MTT test following 48 h of peptide treatment on BHK-21 cells; Figure S4: Antiviral activity of pantinins in BHK-21 cells.

## Abbreviations

The following abbreviations are used in this manuscript:

TOSV	Toscana virus
SBV	Schmallenberg virus
HDPs	Host defense peptides
IC_50_	Half-maximal inhibitory concentration
MTT	Methylthiazolyldiphenyl-tetrazolium bromide
DMSO	Dimethyl sulfoxide
MOI	Multiplicity of infection
DMEM	Dulbecco’s Modified Eagle Medium
FBS	Fetal bovine serum
CMC	Carboxymethylcellulose
CV	Crystal violet
TCID_50_	Tissue culture infective dose
CPE	Cytopathic effect
qPCR	Real-time quantitative PCR
Ct	Cycle threshold
GAPDH	Glyceraldehyde 3-phosphate dehydrogenase
SI	Selectivity index
