# Supplementary material for: Expanding the Antiviral Spectrum of Scorpion-Derived Peptides Against Toscana Virus and Schmallenberg Virus
Source: Pathogens. 2025 Jul 19;14(7):713. doi: 10.3390/pathogens14070713 (PMC12300298; doi:10.3390/pathogens14070713)
Supplement: Supplementary file 1 [file pathogens-14-00713-s001.zip › pathogens-3716336-supplementary.pdf]

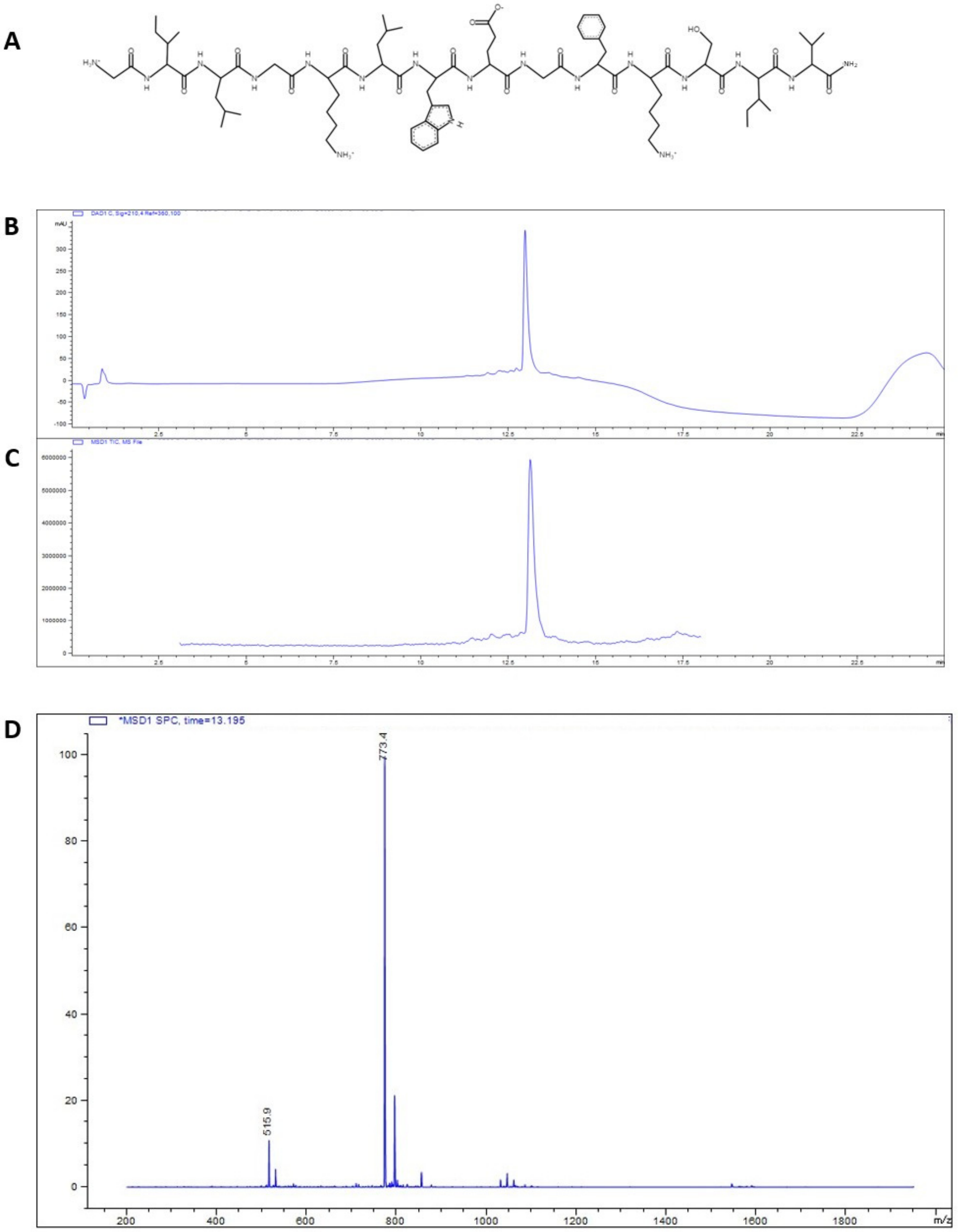

Figure S1. Pantinin-1 characterization. Chemical structure of Pantinin-1 (A), HPLC (B) and MS (C) profiles of pantinin-1. LC-MS analysis were performed using a C18 Waters xBridge (5 µm, 2.1 x 50 mm) column, with linear gradient of 10-80% in CH_3CN/0.05% TFA in water over 10 min at flow rate of 0.2 mL/min. The retention time value of desired product was 13.19 min. (D) MS analysis showed the expected mass for pantinin-1 at m/z: 773.4 $[M+2H]^{2+}$ and 515.9 $[M+3H]^{3+}$.

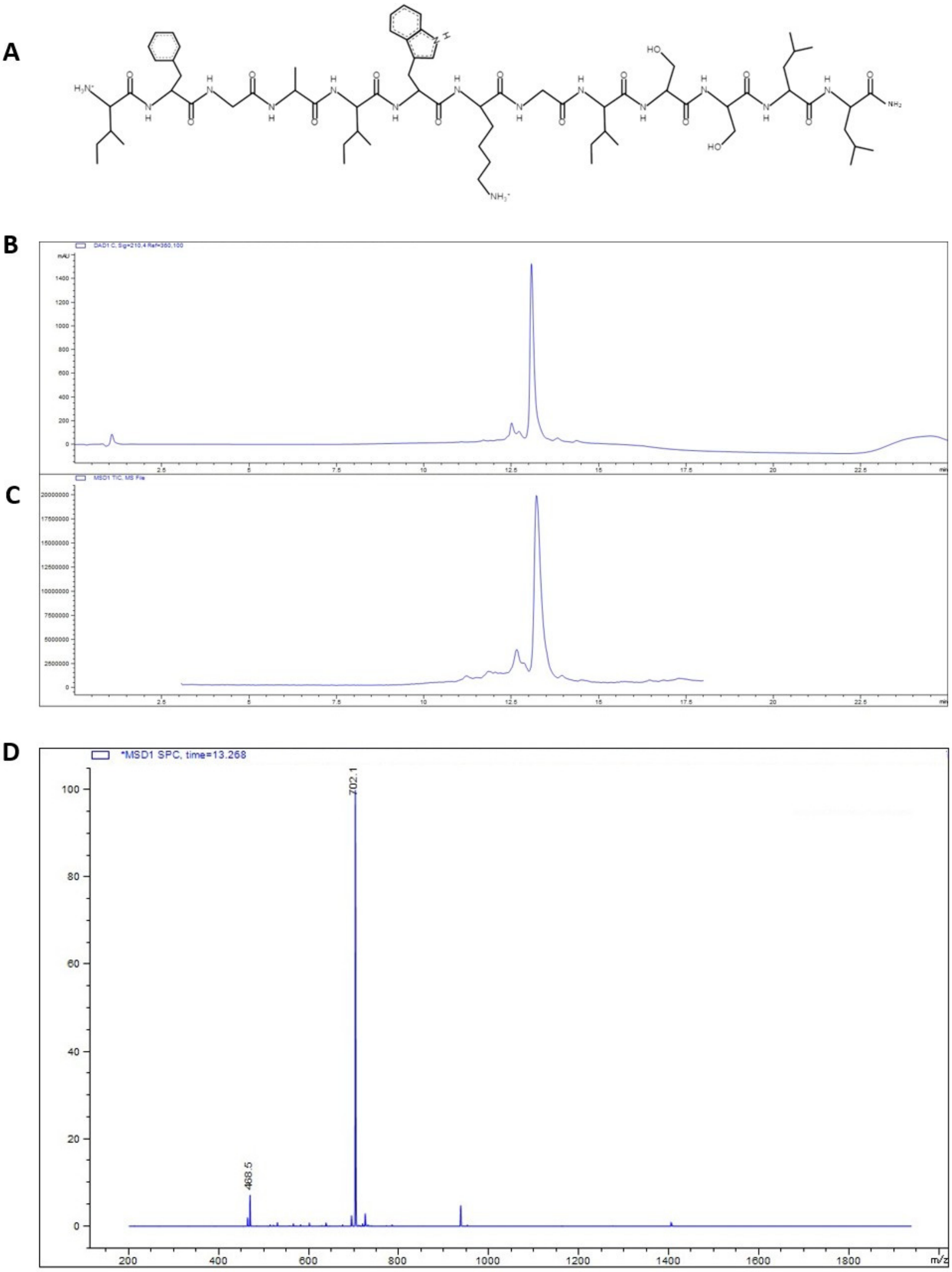

Figure S2. Pantinin-2 characterization. Chemical structure of pantinin-2 (A), HPLC (B) and MS (C) profiles of pantinin-2. LC-MS analysis were performed using a C18 Waters xBridge (5 μm, 2.1 x 50 mm) column, with linear gradient of 10-80% in CH_3CN/0.05% TFA in water over 10 min at flow rate of 0.2 mL/min. The retention time value of desired product was 13.27 min. (D) MS analysis showed the expected mass for pantinin-2 at m/z: 702.1 $[M+2H]^{2+}$ and 468.5 $[M+3H]^{3+}$.

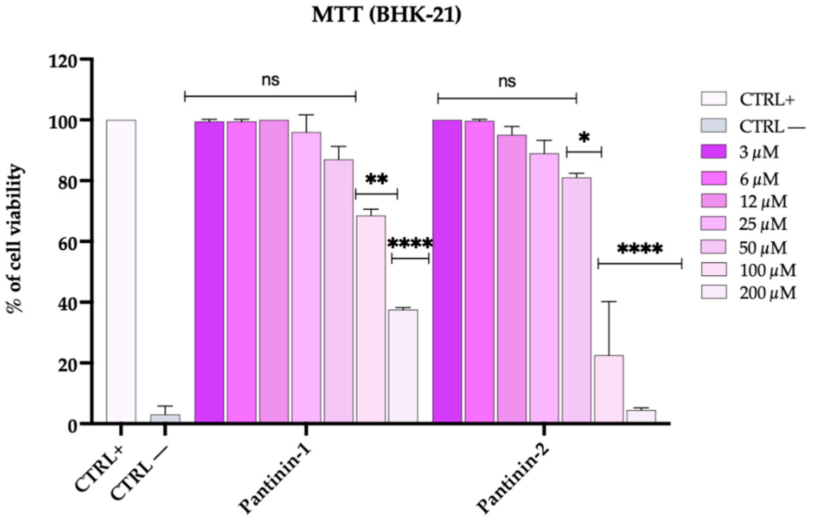

Figure S3. Evaluation of cell viability using the MTT test following 48 h of peptide treatment on BHK-21 cells. The positive control (CTRL +) consisted of untreated cells, whereas the negative control (CTRL —) was represented by DMSO (100%). Two-way ANOVA with Dunnett's multiple comparisons test was performed. Statistical analysis is related to CTRL+. ****p< 0.0001, **p= 0.0021, *p= 0.0332, ns= not significant.

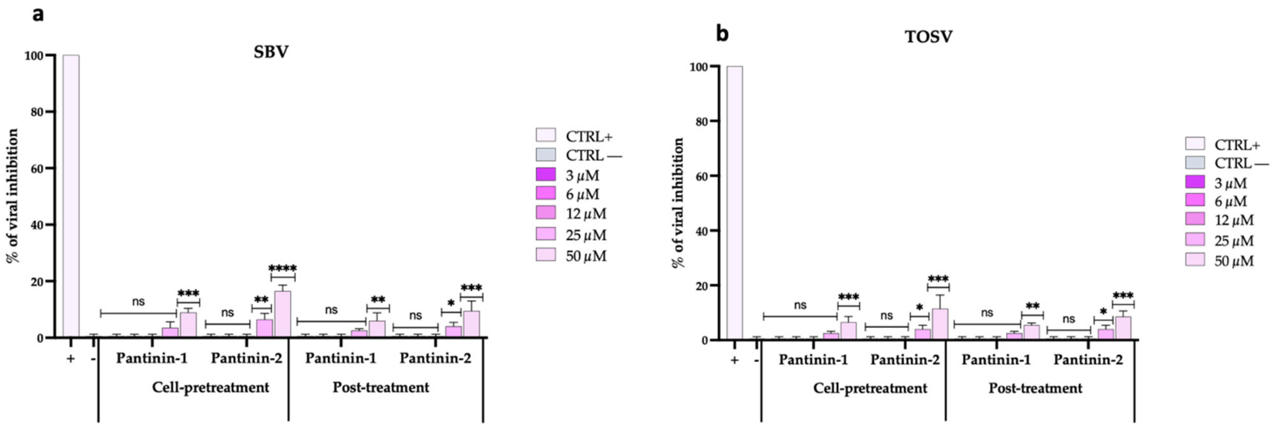

Figure S4. Antiviral activity of pantinins in BHK-21 cells. (**a**) Evaluation of antiviral activity against SBV using the $TCID_{50}$ method in cell pre-treatment and post-treatment assays. (**b**) Evaluation of antiviral activity against TOSV using the $TCID_{50}$ method in cell pre-treatment and post-treatment assays. Two-way ANOVA analysis with Dunnett's multiple comparisons test was performed. ****p< 0.0001, ***p< 0.0002, **p= 0.0021, **p*= 0.0332, ns= not significant.
